# Craniotomy size for traumatic acute subdural hematomas in elderly patients—same procedure for every age?

**DOI:** 10.1007/s10143-021-01548-8

**Published:** 2021-04-26

**Authors:** Daniel Pinggera, Marlies Bauer, Michael Unterhofer, Claudius Thomé, Claudia Unterhofer

**Affiliations:** Department of Neurosurgery, Medical University Innsbruck, Anichstrasse 35, 6020 Innsbruck, Austria

**Keywords:** Acute subdural hematoma, Elderly, Outcome, Surgical technique

## Abstract

Surgical treatment of acute subdural hematoma (aSDH) is still matter of debate, especially in the elderly. A retrospective study to compare two different surgical approaches, namely standard (SC, craniotomy size > 8 cm) and limited craniotomy (LC, craniotomy size < 8 cm), was conducted in elderly patients with traumatic aSDH to identify the role of craniotomy size in terms of clinical and radiological outcome. Sixty-four patients aged 75 or older with aSDH as sole lesion were retrospectively analyzed. Data were collected pre- and postoperatively including clinical and radiological criteria. The primary outcome parameter was 30-day mortality. Secondary outcome parameters were radiological. The mean age was 79.2 (± 3.1) years with no difference between groups and almost equal distribution of craniotomy size. Mortality rate was significantly higher in the SC group in comparison to the LC group (68.4% vs. 31.6%; *p* = 0.045). The preoperative HD (*p* = 0.08) and the MLS (*p* = 0.09) were significantly higher in the SC group, whereas postoperative radiological evaluation showed no significant difference in HD or MLS. A limited craniotomy is sufficient for adequate evacuation of an aSDH in the elderly achieving the same radiological and clinical outcome.

## Introduction

Population aging will lead to a rising number of traumatic brain injuries (TBI) in the eldery, causing major public health and socioeconomic problems [29]. Ten to 20% of TBI patients present with an acute subdural hematoma (aSDH) and the incidence in very old patients has increased over the recent years [14, 37]. The mortality from aSDH in old patients remains high, reaching 40 to 60%, and only 19 to 45% are reported to gain functional recovery [19, 20]. Age over 65 years is associated with poor outcome in many studies and mortality exceedingly rises, if the patient is older than 70 and presents with a GCS score of 9 or less [4, 12, 23, 36]. For aSDH, surgical management was not shown to be an effective treatment in elderly patients with a GCS of 3 to 5 [28].

The current body of literature mainly focuses on younger patients below the age of 65 years, causing low evidence if and how to perform surgery in elderly patients [6, 21, 26]. Surgical treatment of choice for aSDH includes hematoma evacuation via craniotomy or decompressive craniectomy [2, 18, 27]. Parameters to be considered for choosing the surgical approach include, inter alia: radiological parameters, preoperative neurologic status, and associated intracerebral hemorrhage [5, 33]. Lower GCS, greater hematoma thickness, or midline shift correlate with poorer outcome, whereas patients treated with decompressive craniectomy (DC) show either equal or worse outcome [5, 8, 14, 17, 20, 22].

Therefore, operative management of aSDH in the elderly population is a controversial topic, with no treatment guidelines on hand. In older patients, decompressive craniectomy does not show significant benefit after failing maximal medical treatment. One year after decompressive craniectomy, 80% of elderly patients with severe TBI had poor outcome [24]. For all these reasons, clinical decision-making in old patients with aSDH is cumbersome with a heterogenous approach among neurosurgeons as described elsewhere [17, 31, 32]. Besides the decision if to perform surgery at all, the extent of the surgical approach is also a matter of debate, as DC is often associated with serious complications like extraaxial fluid collection, skin-flap-associated subcutaneous hematoma, or external brain herniation [10]. Also, surgical approach and operation time ought to be kept at a minimum to reduce perioperative and postoperative complications [30].

In the present study, we therefore analyzed the influence of craniotomy size on clinical (mortality) and radiological (postoperative hematoma depth and midline shift) criteria. We refer to Jiang et al., who proposes a cut-off value of 80 mm to differentiate between standard (SC, > 80 mm) and limited craniotomy (LC, < 80 mm) [13].

## Materials and methods

All patients aged above 75 years with TBI, who presented to our Department between 01/2000 and 12/2019, were retrospectively reviewed. Of those, only surgically treated patients with isolated aSDH were included in the final analysis. Exclusion criteria were non-traumatic aSDH, subacute/chronic subdural hematoma, further intracranial lesions (epidural hematoma, intracerebral hematoma), or unavailable medical records.

From a total of 564 patients presenting with TBI, 64 patients met the inclusion criteria and were analyzed. All patients were treated in accordance with the Declaration of Helsinki and Austrian regulations. Demographic and clinical data were collected from medical data records including patient age, sex, initial GCS, surgical procedure (standard “large” craniotomy or limited “small” craniotomy), type of trauma (high or low impact trauma), and mortality after 30 days. In addition, presence of anticoagulant or anti-platelet therapy on admission and comorbidities (hypertension, cardiovascular diseases, malignomas) were analyzed. Standard craniotomy (SC) was defined as a frontotemporoparietal craniotomy (> 8 cm) opposed to a temporoparietal craniotomy (< 8 cm, limited craniotomy, LC).

Radiographic parameters collected pre- and postoperatively were hematoma depth (HD) and midline shift (MLS). Hematoma depth was measured in millimeters in the axial plane starting from the inner table of the skull to the innermost boundary of the hematoma. The extent of midline shift in millimeters (MS) was measured also in the axial plane as the perpendicular distance between the septum pellucidum and a line indicating the midline of the skull. Additionally, the maximum anterioposterior length of the bone flap was measured in millimeters on postoperative axial CT scans. Radiological data was gathered from initial CT scans. If a patient was referred from a peripheral hospital, initial imaging was used when available.

To reduce selection bias based on GCS, a subanalysis was conducted in which patients with a GCS of 3 were excluded in the SC group and patients with a GCS of 15 in the LC group, accordingly.

Imaging data were analyzed using Icoserve Advanced Imaging Management (V.1.8.8.34, ITH Icoserve Technology for Healthcare GmbH, Innsbruck, Austria).

Statistical analyses were conducted using IBM SPSS Statistics 24 (IBM Corporation, Armonk, NY, USA). Differences with a *p*-value of less than 0.05 were considered statistically significant. In the univariate analysis, group differences were determined using the chi-square test for dichotomized variables. Continuous variables were analyzed using the independent samples *t*-test and the Mann–Whitney *U*-test, as appropriate. Receiver-operating characteristics analysis was used to calculate the cut-off value for craniotomy size in our series. Multivariate analysis was performed to identify predictive parameters for mortality.

The study was approved by the local ethics committee of the Medical University Innsbruck (Protocol number: AN2020-1115/2020). Due to its retrospective nature and the fact that the study relied on information obtained as part of routine clinical practice, no informed consent was needed.

## Results

### Patient characteristics

In this retrospective study, a total of 64 patients aged above 75 years presenting with an aSDH and requiring surgery were evaluated. Thirty-six (56.3%) were male and 28 (43.7%) were female. The mean age at surgery was 79.2, with age ranging from 75 to 85 years. Mean GCS at admission was 11 (range 3–15). Fifty-four patients (84.4%) received some form of antithrombotic therapy prior to trauma. The remaining patients’ anticoagulation status was either unknown or no record of prior medication was available.

Fifty patients (78.1%) suffered from a low-impact trauma, 14 patients (21.9%) from a high-impact injury.

Hypertension was seen in 46.9% (*n* = 30 patients), cardiovascular diseases in 70.3% (*n* = 30 patients), and history of malignoma in 12.5% (*n* = 8). Comorbidities did not significantly differ when grouped according to the size of bone flap, but a trend is seen towards sicker patients in the group treated with a limited craniotomy (< LC; 80 mm) (Table 1).

**Table 1:** Group comparison of limited versus standard craniotomy (*ns*: not significant)

	Limited craniotomy (LC, < 80 mm)	Standard craniotomy (SC, > 80 mm)	
Number of patients	34	30	
Age (mean)	78.7	79.6	ns
Female (%)	50%	37%	ns
GCS (mean)	12	7	*p* = 0.013
Size of bone flap (mean)	52.7 mm	108.6 mm	*p* = < 0.01
Hematoma depth preoperatively (mean)	15.2 mm	19.4 mm	*p* = 0.04
Midline shift preoperatively (mean)	9.9 mm	14.7 mm	*p* < 0.01
Hematoma depth postoperatively (mean)	4.8 mm	6.2 mm	ns
Midline shift postoperatively (mean)	4.4 mm	4.7 mm	ns
Hypertension	32.8%	14.1%	ns
Cardiovascular diseases	43.8%	26.6%	ns
Malignomas	7.8%	4.7%	ns
Percentage reduction of hematoma depth (mean)	71.5%	63.4%	ns
Percentage reduction of midline shift (mean)	56.1%	54.2%	ns
Mortality within 30 days	23.1%	52.0%	*p* = 0.045

## Radiological data and surgical approach

Mean hematoma depth preoperatively amounted to 17.2 mm (range 8–32 mm), and mean midline shift was 12.1 mm, ranging from 0 to 30 mm. Two patients presented with a hematoma depth below 10 mm but were treated surgically due to present midline shift and severe neurological symptoms. Measurement of radiological parameters postoperatively showed a mean residual hematoma depth of 5.5 mm (range 0–21 mm) and a mean residual midline shift of 4.5 mm (ranging 0–13 mm).

Mean size of the bone flap was 78.9 mm, ranging from 27 to 161 mm. For better comparison to the literature, a cut-off value of 80 mm was defined [13].

**Fig. 1:**
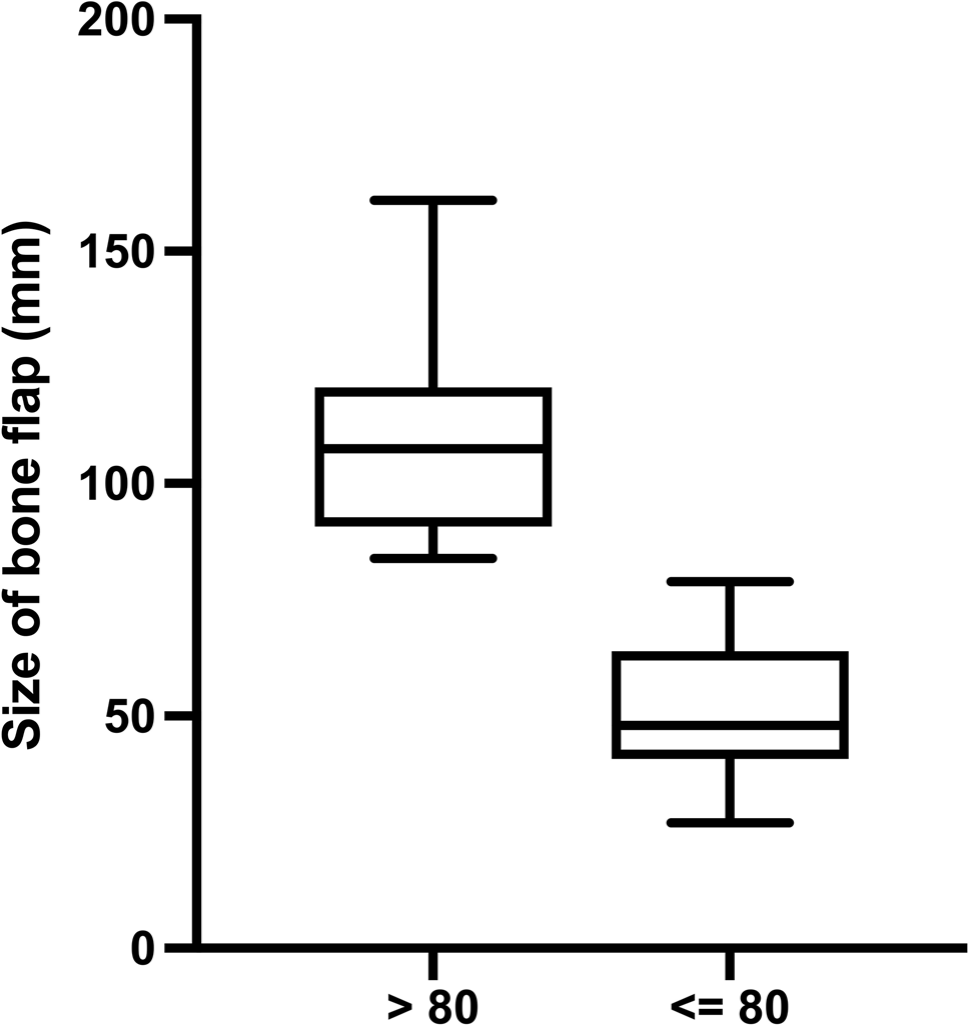
Distribution of size of bone flap between LC and SC (median + IQR)

Grouped according to the size of bone flap, 30 patients (47%) were treated with a limited craniotomy (< LC; 80 mm) and 34 patients (53%) with a standard craniotomy (SC; > 80 mm). This resulted in an average flap size of approximately 5 cm in the LC group and of approximately 11 cm in the SC group (Table 1 and Fig. 1).

Twelve SC patients (18%) received a decompressive craniectomy defined as an extensive craniotomy with the bone flap left out. Age and gender were distributed equally among groups, same as postoperative hematoma depth and midline shift. Preoperative depth of hematoma and midline shift were significantly higher in the *SC group*, whereas GCS was significantly lower in this group.

Logistic regression analysis was created using the following variables: GCS, size of bone flap, hematoma depth, and midline shift. The model was then optimized using backward stepwise conditional elimination. In the resulting model, none of the parameters showed statistical significance.

### Outcome

Outcome was analyzed as trauma-related mortality within 30 days, which accounted to 36% (18 patients) overall. There was a significantly higher mortality in the *SC group* with no benefits in regard to radiological parameters. None of the patients had to undergo second surgery for residual hematoma (Table 1). Within the *SC group*, 2 patients with decompressive craniectomy died within 30 days.

### Subanalysis

Excluding patients with a GCS of 3 in the SC group and patients with a GCS of 15 in the LC group led to a more homogeneous distribution of GCS between groups with a mean of 10 (SC group; *n* = 21) and 12 (LC group; *n* = 25), respectively. Mortality was comparable at 25% and 30% and no other parameter showed a statistically significant difference (Table 2).

**Table 2:** Group comparison limited versus standard craniotomy in the selected analysis, excluding patients with a GCS of 3 in the SC group and GCS of 15 in the LC group (*ns*: not significant)

	Limited craniotomy (LC, < 80 mm)	Standard craniotomy (SC, > 80 mm)	
Number of patients	25	21	
Age (mean)	79.2	79.5	ns
Female (%)	56%	47%	ns
GCS (mean)	10	12	ns
Size of bone flap (mean)	52.6 mm	104.4 mm	*p* = < 0.01
Hematoma depth preoperatively (mean)	15.3 mm	18.4 mm	ns
Midline shift preoperatively (mean)	9.8 mm	13.6 mm	ns
Hematoma depth postoperatively (mean)	5.1 mm	6.8 mm	ns
Midline shift postoperatively (mean)	3.9 mm	4.2 mm	ns
Percentage reduction of hematoma depth (mean)	77.3%	64.5%	ns
Percentage reduction of midline shift (mean)	59.9%	59.8%	ns
Mortality within 30 days	25.0%	30.0%	ns

## Discussion

While evacuation of aSDH in the general trauma population is usually performed via an extensive craniotomy or decompressive craniectomy more than 10 cm in size, a small craniotomy may suffice in elderly patients. The results of this first study investigating the influence of craniotomy size in patients aged above 75 years with aSDH demonstrate that a craniotomy sized 80 mm or less may achieve a sufficient radiological result with small residual hematoma depth and MLS not requiring revision surgery.

Despite an aging population with a consecutively rising number of TBIs, sufficient data and treatment guidelines are lacking [29]. Therefore, the choice of the surgical procedure in elderly patients is still based on data generated in younger patients or on the surgeon’s expertise. Important factors influencing indication for surgery in the elderly include age, midline shift, hematoma thickness, and presence of anticoagulation therapy [31]. Interestingly, in patients on oral anticoagulation medication and aged above 80 years, those radiological parameters do not seem to influence outcome and an oral anticoagulant therapy regimen is exceedingly common as seen in our data (84%) and the literature [37, 38]. This also comes in hand with an increasing number of patients presenting with delayed neurological deterioration. Minor traumatic events in elderly patients on anticoagulant therapy can easily cause aSDH, resulting in a delayed appearance of symptoms with initial unremarkable neurological status [2, 14]. Given the results of an online questionary, almost half of neurosurgeons choose a large osteoplastic craniotomy when performing surgery, in contrast to 13% considering a small osteoplastic craniotomy or 28% performing a decompressive craniectomy [31]. The latter comes with drawbacks including subdural effusion, leakage of cerebrospinal fluid, or external herniation [9, 10]. However, there is a paucity of evidence in the literature regarding the best surgical strategy for aSDH for the elderly and surgical decision-making is often haphazard. Additionally, the discussion of craniotomy versus decompressive craniectomy is still controversial, same as the size of craniotomy [15, 27, 34]. Data obtained in the prospective RESCUE-ASDH trial are long-awaited [16].

In the elderly, neurosurgeons try to achieve a balance between largest craniotomy possible, while avoiding concurrent drawbacks of decompressive craniectomy [7, 18]. A small series of 44 patients aged above 75 described a low mortality, when patients were conscious and without antithrombotic medication prior to surgery [25]. The type of surgery, however, was not reported. Similar to our results, another series of 62 patients aged above 65 years found a mortality of 37% [1]. Most of the patients (86%) were treated with a craniotomy. In addition to the younger age, midline shift was higher and patients with intracerebral contusions were included as well, making comparison difficult [1]. Corresponding to our findings, no significant differences in hematoma depth and midline shift pre- and postoperatively in respect to outcome in a cohort mainly treated with craniotomy were reported [3]. Yet, no further details about the size of the craniotomies are presented, neither is elsewhere in the literature. As no second surgery or re-operation had to be performed in our series, we conclude that there is little risk for secondary brain swelling in this population. Also, in the overall and selective analysis, a LC with an average flap size of approximately 5 cm shows comparable postoperative radiological and clinical outcome.

Age and low GCS are still the major determinants of outcome after brain injury with increasing mortality with higher age [3, 28, 35]. Very old patients with an initial GCS of 3–5 have an unfavorable outcome and higher mortality as compared to those with a GCS of 6–15 [25, 28]. In addition, patients aged above 80 years will die in 50% after critical illness with only one-fourth recovering to pre-hospital function [11]. Still, tendencies to perform surgery are high, even in the elderly [1, 25, 28, 37]. Also in our study, patients with a low GCS and SC or DC showed a high mortality rate in univariate analysis, but not in multivariate analysis. A possible explanation may be found in the sample size and the collinearity of the radiological parameters. The subanalysis after excluding the very poor and the very good patients investigated comparable patients according to their initial GCS. In these 44 patients (20 vs. 24), a small “5 cm” craniotomy allowed a sufficient hematoma evacuation.

Our study is within the range of mortality rates reported in the literature for surgical evacuation of aSDH in the elderly population. Studies that had an even older age as inclusion criterion reported worse mortality rates, except for Won et al., who found an initial 27% mortality rate in patients over 80 years of age who underwent surgical evacuation for aSDH [37]. The type of surgery, however, was not mentioned. All in common are little case numbers, hampering comparison [3].

Several limitations need to be acknowledged in our study, being retrospective the most obvious one. Furthermore, our cohort probably represents a selection of patients who were judged to have good chances of favorable outcome by the attending neurosurgeons and therefore received surgery. Additionally, clinical outcome was assessed as mortality within 30 days, excluding patients dying thereafter. Last, group allocation was based on the decision of the attending surgeon and not on random allocation.

In summary, patients aged 75 years or above with aSDH do not seem to be at risk for postoperative brain swelling and hematoma evacuation can be achieved via a small craniotomy. This may minimize surgical trauma, and reduce operative time and wound complications. Further studies should be performed to confirm our results. It needs to be kept in mind that the vast majority of patients receive anticoagulative or antiaggregative medication.

## Conclusions

Neurosurgeons tend to apply larger craniotomies for aSDH even in the elderly in case of low GCS and large HD and MLS. Limited craniotomies with an average size of 5 cm, however, seem to be sufficient and feasible in patients aged above 75 years to evacuate aSDH, achieving comparable surgical decompression.

## Data Availability

Data transparency was confirmed.
